# Regulatory barriers to equity in a health system in transition: a qualitative study in Bulgaria

**DOI:** 10.1186/1472-6963-11-219

**Published:** 2011-09-17

**Authors:** Boika Rechel, Clare M Blackburn, Nick J Spencer, Bernd Rechel

**Affiliations:** 1 Norwich Medical School, University of East Anglia, Norwich, UK; 2 School of Health and Social Studies, University of Warwick, Coventry, UK; 3 European Observatory on Health Systems and Policies, London School of Hygiene & Tropical Medicine, London, UK

## Abstract

**Background:**

Health reforms in Bulgaria have introduced major changes to the financing, delivery and regulation of health care. As in many other countries of Central and Eastern Europe, these included introducing general practice, establishing a health insurance system, reorganizing hospital services, and setting up new payment mechanisms for providers, including patient co-payments. Our study explored perceptions of regulatory barriers to equity in Bulgarian child health services.

**Methods:**

50 qualitative in-depth interviews with users, providers and policy-makers concerned with child health services in Bulgaria, conducted in two villages, one town of 70,000 inhabitants, and the capital Sofia.

**Results:**

The participants in our study reported a variety of regulatory barriers which undermined the principles of equity and, as far as the health insurance system is concerned, solidarity. These included non-participation in the compulsory health insurance system, informal payments, and charging user fees to exempted patients. The participants also reported seemingly unnecessary treatments in the growing private sector. These regulatory failures were associated with the fast pace of reforms, lack of consultation, inadequate public financing of the health system, a perceived "commercialization" of medicine, and weak enforcement of legislation. A recurrent theme from the interviews was the need for better information about patient rights and services covered by the health insurance system.

**Conclusions:**

Regulatory barriers to equity and compliance in daily practice deserve more attention from policy-makers when embarking on health reforms. New financing sources and an increasing role of the private sector need to be accompanied by an appropriate and enforceable regulatory framework to control the behavior of health care providers and ensure equity in access to health services.

## Background

Bulgaria, a post-communist country with a population of 7.50 million people in 2010 [1] situated in South-East Europe, joined the European Union (EU) in January 2007. In the late 1990s, Bulgaria began embarking on major reforms of its health system. These included introducing general practice, establishing a health insurance system, reorganizing hospital services, and setting up new payment mechanisms for providers [2]. Up to 1998, Bulgaria's health system had been mainly financed through general taxation and was characterized by a number of weaknesses, including underfunding, a focus on curative and hospital services, and lacking incentives for improving quality and efficiency. The introduction of social health insurance in 1998 aimed to increase the resources available for health care, improve efficiency, and regulate the scope and quality of health services [3,4]. The National Health Insurance Fund (NHIF) was established in 1999 as an autonomous institution responsible for collecting health insurance contributions, paying health care providers and guaranteeing access to health services for the insured population [2]. Health insurance contributions were initially set at 6% of income in 1999, split between employer and employee at an 80:20 ratio, and reached 8% and a ratio of 60:40 in 2009 [2,4]. General practitioners (GPs) are paid on a capitation basis according to the number of registered persons in their practice.

In addition, patients pay user fees for every consultation with a GP or a specialist, set at 1% of the minimum monthly salary (110 Euro in 2008), and per day of hospitalization, set at 2% of the minimum monthly salary, for a maximum of 10 days per year. Children under 18 years of age, patients with certain chronic conditions and other vulnerable groups of the population are exempted from user fees [4].

This article, which forms part of a wider investigation of access to child health services in Bulgaria, describes participants' views of challenges to regulation, as expressed by users and providers of health services, as well as policy-makers. This issue has emerged in our study as one of the key problems associated with access to child health services and one that has been neglected in the academic literature. While informal payments in Bulgaria's health sector have been described previously [5,6], hardly any investigations have so far been undertaken in Bulgaria or elsewhere in Central and Eastern Europe about the broader challenge of regulating provider behaviour in health systems in transition. Our study aims to start filling this gap.

The pursuit of equity in access to health services is an explicit objective of many health systems [7]. However, in most health systems access to health services is inequitable, with more and higher quality services for the well-off than for disadvantaged groups of the population who generally need health services more often but are unable to obtain them [8].

This paper explores regulatory barriers to equity in accessing health services. Key regulatory functions of governments in the health system include standard-setting, monitoring and enforcement [9]. These functions are usually exercised through different regulatory bodies at national or regional level, including the Ministry of Health, third party payers, agencies for quality of care and patient safety, and professional associations. Examples of regulatory instruments that we have found to be of particular relevance to the Bulgarian context are given in Table 1.

**Table 1:** Examples of regulatory instruments and relations relevant to the Bulgarian context

Between government/financing agencies and patients	• Systems of financing and decisions on the statutory health system • Population coverage: setting out the breadth (proportion of population covered), scope (range of benefits covered), and depth (proportion of benefit costs covered) of benefits
**Between financing agencies and service providers**	• Provider payment mechanisms • Harnessing the market in contracting for health services • Access of providers to health care markets and financing agencies

**Between service providers and patients**	• Access of patients to health care providers • Patient rights, litigation, criminal damages • Mandatory reporting by providers of quality information, price lists and performance • Self-regulation of providers through professional codes of practice and voluntary or mandatory accreditation schemes

Source: Adapted from [9-11]

As illustrated in Table 1, a number of regulatory instruments are being used in health systems to define the basic parameters of the system and ensure its functioning. In this paper, we pay particular attention to those aspects of regulation that were identified by our respondents as posing obstacles to equitable access to child health services in Bulgaria.

## Methods

Our study followed a case-study approach using multiple methods and sources of data: qualitative in-depth interviews, an analysis of regulatory documents, and a review of the literature and existing epidemiological data. The results of our documentary analysis have been reported elsewhere [12], and the qualitative research methods have been described earlier [13,14]. In order to increase the validity of our findings, respondents included many different stakeholders: policy-makers, health care providers, and users of services, from both rural and urban areas (two villages, one town of 70,000 inhabitants, and the capital Sofia). Furthermore, considering those likely to face particular challenges with regard to access to services, the sample included users from the country's Roma minority, as well as parents and other stakeholders concerned with disadvantaged children. A total of 50 interviews were conducted by the lead author in Bulgarian and English. Participants were recruited by the lead author from the following groups:

• health care providers working with children (n = 13), including GPs, paediatricians, nurses, a dentist, and a manager of a children's institution;

• parents and carers of young children (n = 12), including representatives of ethnic minorities and parents of children with long-term conditions;

• policy-makers (n = 10), including representatives of government departments, the NHIF and international organizations;

• other stakeholders (n = 15), including organizations working with the Roma population, with institutionalized and disabled children, and experts from academic institutions.

Recruitment was purposive and hardly any of those approached refused to participate. The interviews were conducted during four periods of field work between October 2005 and April 2007. The interviews were in-depth, broadly based on a topic guide that differed for providers, policy-makers and users and is available from the authors upon request. All respondents were given detailed information about the study and informed consent to participate was obtained. Interviews were tape-recorded with the permission of the participants and transcribed verbatim (n = 42), or otherwise detailed hand-written notes were taken (n = 8). We ensured participants' anonymity and, where requested, confidentiality. Thematic analysis was undertaken which followed the principles of grounded theory [15]. In the interpretation of the interview material, we paid particular attention to the role of the lead author and interviewer as a physician coming from Bulgaria and having worked outside the country for some time, and how this might have influenced the course and content of the interviews. We derived themes from the data inductively, guided by our previous knowledge, and looked for deviant cases and evidence supporting alternative explanations. Ethics approval was obtained from the School of Health and Social Studies at the University of Warwick and from the Bulgarian Sociological Association.

## Results

### Health insurance contributions, official co-payments and informal payments for health services

From many interviews it appeared that participants were not fully informed about the rationale of the health insurance system and why patients had to pay fees for outpatient consultations after having paid health insurance contributions. The perception was common that the personal insurance contributions ought to be accumulating in an individual fund for use by that person in case they needed health care. Many participants noted the lack of clarity surrounding insurance contributions and co-payments and difficulties in obtaining information about how the system is functioning:

"I don't really understand the health insurance fund. My husband has not been to a doctor probably since the reform took place [in 1998]. However, he is paying [insurance contributions]. Finally, when he goes to the doctor, he has to pay again. The other money, where did it go, it is not clear. I think, it needs to be accumulating somewhere, doesn't it? Last time they told us, he has used the money without going to the doctor, which cannot be true, can it? Nobody explains anything to you, if you ask." (immigrant mother of three children)

A number of respondents did not seem to have been informed about the principles of solidarity and risk-sharing which underpins any health insurance system. They perceived health insurance contributions as unfair because an individual's contributions did not necessarily match their use of health services.

"Unfortunately, those who pay the least [contributions], use services the most." (policy-maker)

"The insurance payments should be registered on a card so that everyone knows what amount of money they have at their disposal for medical services. At present, the person who pays contributions based on 1000 Lev monthly income, and the person who is insured on 160 Lev income, they both receive the same service. This, I think, is not normal." (GP, formerly specialist paediatrician)

Our respondents described informal payments as being common in both outpatient services and the hospital sector. However, several participants viewed them as an acceptable survival strategy in the current socio-economic environment:

"The social problems and transformations have a very serious influence on health professionals. People, understandably, don't manage to separate professional standards and norms from personal interest, which is absolutely understandable in these times when one needs to provide for one's family to survive." (policy-maker)

Informal payments in health care need to be seen in their societal context. Another participant, whose husband was working as a surgeon, perceived charging patients for surgery as a necessity, as doctors themselves were "victims" of over-charging for other services (she gave examples of a car mechanic and an architect). In her view, taking money from the "same people" when they came in need for surgery was justifiable, because a surgical operation was no less complex than fixing a car or designing an architectural project.

Charging official fees for child health services (for example for laboratory investigations or for dental treatments) and informal payments for maternal and child health services were perceived by the participants as contradicting the government's formal commitment to prioritizing maternal and child health:

"They [the health care providers] know very well that everybody would do everything for their child and would give everything and sometimes they take advantage of that, which I think is unfair. It is true that there is not enough money for everything, it is never enough, but for the children's health, they should not take advantage in this way." (mother of a six-year-old child)

Although many participants believed child health services to be less prone to improper practices than adult services, most interviewees identified informal payments as a persisting problem in accessing health services:

"Another problem that continues to exist, and it is not related to the Roma only, is that the family physicians and not only they, the hospitals as well, continue to take money which they are not supposed to take according to the law." (NGO representative, mother of a two-year-old child)

Payments for maternity services were described as being common. One nurse described her experience of paying a fee in order to ensure good care for her delivery. As she was a health professional herself, she paid a fee that was lower than usual, a practice that seems to be common among health professionals:

"We paid for the delivery. At that time, five years ago, it was 300 Lev [150 Euro], it wasn't little money at all. [...] However, we paid it gladly in order to receive good service." (nurse, mother of a six-year-old child)

According to one policy-maker, a pregnant woman without health insurance who is admitted to hospital for delivery has the option of paying her health insurance retrospectively, leaving a maximum of three unpaid monthly contributions. She reportedly needs to arrange this in the period between admission and discharge. Another participant said that women pay if they choose to receive additional or different services, for example a particular physician to be present, or to have epidural anaesthesia or elective Caesarian section. Several of our participants reported cases of inducing birth, so that the date of delivery coincided with a particular doctor's duty shift.

As was described above, children under 18 years of age are officially exempt from user fees. However, some of the users, providers and NGO representatives in our study held that, in practice, user fees were still being charged for consultations with GPs, confirming the finding of other studies that under-the-counter payments and user fees are often difficult to distinguish [16,17].

"In the beginning, when the health reform started, the consultations for children had to be free, but until things settled, they charged user fees for children, even for them. It depends on the physician. Maybe simply some of them had not yet understood fully how things were." (mother of a six-year-old child)

If parents are not well informed and have less bargaining power, they seem to be more often asked to pay user fees and other charges. People with low levels of education and ethnic minorities seem to be more likely to be victims of lack of information:

"It happens very often, including in the Roma quarters, but not only there, that physicians charge user fees also for children. This happens, although it seems to me that people are increasingly more informed and know that it shouldn't happen. We have heard such complaints. Physicians are obliged to display information on a visible place which health-insured persons are exempted from user fees, there are different categories. This is not being done." (NGO representative)

Uncertainty arises from the co-existence of official co-payments, seemingly common informal payments, and frequent changes in regulations specifying for which services co-payments are required:

"There are some other kinds of fees. For children, in principle the investigations are for free. But if I go with [my son] to the laboratory for them to take a blood and urine sample from him, then I pay 2 Lev at the laboratory. The investigation is free, but this is a different kind of fee." (father of two children)

### Regulation of the private sector

The Public Health Act from 1971 was amended in 1991 to legalize private medical and dental practice. There are increasing numbers of private dental surgeries, pharmacies, single and group practices for primary care (general practice), specialist medical practices, diagnostic laboratories and private hospitals. The private sector in Bulgaria can be defined as those providers who do not hold contracts with the NHIF and are paid for their services directly by patients. Similar to some other countries in Europe, many specialists working in public hospitals in Bulgaria also have their own private practice, and mixing public and private commitments seems to be common, with doctors using hospital equipment and encouraging patients to use their private practices. Several participants, including both providers and users of services, described cases of doctors recommending patients seemingly unnecessary treatments. Offering treatments apparently guided by financial interest rather than clinical need was perceived as being particularly common in the private sector, which is not regulated by the NHIF. Furthermore, private services were associated by our respondents with over-diagnosing in order to induce need for investigations and treatment. One father talked about his experience of seeing a private paediatric allergy specialist for his son, who was charging high fees for tests which were also available under the NHIF at much lower rates. The private doctor wrote "a long prescription" of drugs that were "for someone very poorly, with severe allergy". A subsequent consultation with a GP and tests under the NHIF showed that the child did not suffer any allergy.

Another participant expressed concerns about the practices in the private sector, such as performing seemingly unnecessary surgical operations:

"Unfortunately, the alternative is the private sector which is also very risky, because very serious irregularities occur there as well." (NGO representative)

Reportedly referring children to certain specialist providers or certain pharmacies are further examples of practices affecting access to child health services:

"Led by financial interests, the GPs refer the parents and the children always to the same specialist providers. The parents should have the right of choice and judgment, rather than the family physicians to impose their [...] selected specialists." (policy-maker)

The participants considered appropriate prescribing practices to be an indication of the professional qualification of physicians. However, other factors that influence prescribing practices were also identified, such as incentives from the pharmaceutical companies producing the drugs, and material interests from the sales of drugs at particular pharmacies.

"There are also monetary transactions between the firms which release the antibiotic and the people who prescribe it. Everything is a question of material, so-called, material incentives, regretfully, which contradicts all ethical principles." (neonatologist)

"The personal physicians work with certain pharmacies. They tell the patients where they should go to buy expensive antibiotics, expensive drugs, while there are cheaper ones with the same effectiveness from our [local producer] Pharmachim." (immigrant mother of three children)

### Perceived causes of failures of regulation

#### The magnitude and pace of change

Policy-makers and doctors described the speed of change as a major challenge. The system based on general practitioners was introduced suddenly, with many doctors given no choice and having had no time to adapt or to understand the meaning and purpose of health reforms.

"Within six months. A **total **change. This lack of evolution in fact left the doctors for a long time..., the doctors themselves didn't understand what was happening, let alone ordinary people." (policy-maker)

"For us, this [the reform] was very stressful because we were not given any choice in the year 2000. You were told 'Until June you have a job at the polyclinic as a state employee. From 1^st ^of July you set-up private cooperatives and start working as general practitioners.' Everyone of us is a specialist. For example, I am a paediatrician. And suddenly from a paediatrician I have to transform myself into a general practitioner and to work with elderly patients. And up to that moment, for 16 years, I have dealt only with children. That was really stressful." (GP, formerly specialist paediatrician)

#### Lack of participation and sense of ownership of the reforms

Another concern expressed by our participants was that the reform had not resulted from a broad public debate and a process of participatory decision-making, but rather imposed top-down in a very short period of time. It seems to have taken a long time for managers and health care providers to adapt to the changes, but the adaptation has been even more difficult for users of health services:

"[I]n our country it has not resulted from an evolution of public thinking, of perceptions of the people about the system of public health. In our country it [the reform] was imposed in a purely administrative way as a model of the health system in a very short period of time. At the moment this is the sixth year since the introduction of the reform. And the public opinion still does not accept it." (policy-maker)

One NGO representative noted that the lack of participation and consultation in developing health strategies has undermined the effectiveness and sustainability of health reforms.

"I think that there isn't good planning and good strategy because obviously these strategies are not being developed by a wide circle of specialists or a wide circle of interested institutions, which would make the system, so that health care is real." (NGO representative)

#### "Commercialization" of medicine

Some participants believed that health reforms may have benefited some private enterprises, such as pharmaceutical companies, but that they have failed to benefit the population.

"I don't know what the health policy is, and what is happening, but it is not in the interest of the people. It may be in the interest of business, or what. But I think it is not in the interest of the people." (village council worker)

Negative attitudes were in particular expressed towards the "commercialization" of health care. The shift towards a market economy and competition in the health sector was viewed by some participants as "trade with peoples' health".

"Everything became totally mercantile. That is the biggest disadvantage of the reform. The physicians became 'single merchants', the hospitals became 'trading companies'. Human health should be the first priority. [...] Now hospitals are required to present a financial plan in the first place. Nobody is interested whether patients have died there, or whether the treatment is effective there. " (hospital paediatrician)

Another doctor believed that the market principle is in general unsuitable for the health sector. In her opinion, ensuring the best quality of care should receive a higher priority than cost-containment:

"They turned the GPs into guards of the Fund. For me personally, it is a disadvantage that we have embraced the market mechanism with regards to health care. Because the market mechanism, if we talk about goods-and-money relationships, indeed it has proven its advantages. However, concerning trading with health, you can't put that on market principles. [...] People remain under the impression that this reform was done with the main aim of minimizing health care expenditure rather than maximizing the quality of health care." (GP)

#### Inadequate financing of the health system

The apparent high prevalence of direct out-of-pocket payments to providers in Bulgaria needs to be viewed in the context of persisting funding shortages in the health system, resulting in low salaries of health workers and inadequate financing of health facilities. Several GPs participating in our study complained about children's exemption from user fees. In their view, the capitation fee of one Lev [0.50 Euro] per month was insufficient compensation for the time and costs involved in providing health services for children:

"I am responsible for the acute illness of a child. I see her three, four times, because for an infectious disease she cannot be examined just once and I cannot close the case with that. There is a need for a follow-up examination. Especially in infancy and early childhood, the matters are very serious. Children don't pay user fees, they are exempted, and actually I receive for the whole month only one Lev capitation fee [...] which should also cover the labour of the nurse, and the consumables" (GP)

Other categories of patients are exempted from user fees as well, and this situation does not seem to be acceptable to GPs, who do not receive payment for consulting these patients. As one GP put it, these exemptions were made at the expense of doctors who are not compensated by the state for the services they provide for exempted patients.

"Somehow they make social policy at the expenses of the working people, of the doctor. This is what makes me very angry. Now I am a private practitioner. It cannot be that the children and a mass of people are exempted from user fees. And if the state has exempted them, I should receive money for examining them. I examine them numerous times without receiving any money." (GP)

This quote indicates that exemptions from user fees might not be welcomed by all GPs. The exemptions seem to present disincentives for GPs to register on their lists a large number of children and chronically/severely ill adults, who need care and follow-up, but are not officially required to pay user fees for their consultations with the doctor.

#### Weak enforcement of regulations

Our participants recognized that not all legislation was being fully implemented, which created a discrepancy between intentions for ensuring accessible and high-quality services and the reality of service provision:

"The problem is that in Bulgaria very often documents are enacted, but their implementation is not required or undertaken by the relevant institutions. If you review the legislation, the situation in Bulgaria most probably would appear very good. However, there is a lack of regulatory acts for implementation, systems of monitoring and control, and systems of evaluation, which is a very serious problem." (policy-maker)

One policy-maker mentioned the lack of continuity in implementing certain reforms and the slow and sporadic character of different activities, which depend to a great extent on the attitudes of individuals in key positions:

"Unfortunately, what has been decided in the framework of the reform is rarely implemented, or the activities are very slow or sporadic. Or something may be undertaken, it is being considered for 2-3 months, it is talked about, and after that it is stopped, if the responsible person in the ministry or the institution is not interested to continue the efforts until they have reached a concrete solution." (policy-maker)

Many respondents were skeptical about the chances of overcoming the problem of informal payments. They perceived this practice as being deeply rooted in the country's culture and exposures of cases of corruption in the media were believed not to have any real consequences:

"This is a problem which I think will not be solved soon. They [informal payments] have existed for a long time and are deeply rooted in our culture. There are many problems with this kind of payments. They are known officially. They are published again and again in the press: 'this hospital, these physicians', but the physicians appear in public and say 'that is not the case'. There is only talk, but nothing is being undertaken and nothing will change." (NGO representative, mother of a two-year-old child)

Interestingly, in some cases non-compliance with regulations on behalf of providers is for the perceived benefit of patients, and it may widen access to care. For example, it became apparent from the interviews that children who do not hold Bulgarian passports, or do not have a unique citizen's number (EGN), are not entitled to receive health services. Their parents have to pay for health care privately. A mother of a child who was a foreign citizen described her experience as follows:

"We have a problem with our oldest daughter, because she is not a Bulgarian citizen. When we go to see a doctor now, we have to pay for her as for an uninsured person. She doesn't have an EGN, she doesn't have anything like that, she is nobody. However, our doctor has never refused to see her and she doesn't even take any money. If it was someone else, I would have to pay 10 Lev per examination, like for an uninsured person. We wrote to the president about this, we told him we pay for everything and it's very difficult for us." (immigrant mother of three children)

One interviewed GP confirmed that this issue is not resolved administratively, but some physicians consult children on a good-will basis, although they are not reimbursed for the consultations by the NHIF.

"I think access is not arranged in any way for foreign citizens. But quite often, they are examined on a good-will basis." (GP)

According to Order No. 2 of the Ministry of Health from 2005, foreign citizens pay for medical services according to prices defined by the respective health facility [18]. The above interviews were conducted in April 2006. Subsequent amendments to the Health Insurance Act in force since January 2007 introduced a new paragraph (article 33 par. 3), specifying that "foreign citizens or persons without citizenship with permitted permanent stay on the territory of the Republic of Bulgaria" shall be covered by the NHIF [4].

In addition, it appeared from the interviews that patients are sometimes hospitalized for social reasons and not according to medical need. Different examples were provided by both providers and users of services. Whether a child is treated in hospital or on an ambulatory basis may depend on the parents' ability to pay for drugs and procedures (such as injections). In primary care, parents pay the full price of drugs in pharmacies, but if the child is hospitalized, the drugs are provided for free. Inability of parents to pay for outpatient drugs can be a reason for hospitalization, so that the child can be treated without putting a financial burden on the family:

"We hospitalize [...] when the parents for financial-economic reasons cannot afford the luxury of treatment with injectable drugs as outpatients [...], which are not at all cheap." (paediatrician, outpatients facility)

#### Insufficient information about the health reforms

A recurrent theme from the interviews was the need for information about patient rights and services covered by the statutory system, something that was perceived as a necessary condition for people to be able to seek appropriate care. The participants indicated that, during the early stages of the health reform, not enough information was provided to the public about the changes in the provision of health services:

"There were lots of changes and very little information, very little information. The National Health Insurance Fund created some websites, telephone numbers and so on, but in reality this information does not reach the population. Who has the opportunity to use the internet to check? Simply a very powerful information campaign should have been run in parallel to all this transformation, by the Ministry of Health, by the National Health Insurance Fund, about every change that was happening in the health system." (NGO representative, mother of a two-year-old child)

Users often mentioned that they were not very clear about their rights and that information was difficult to come by:

"We are really not very clear about our rights. I think there is not enough information. There has to be more, especially in the regions, the small towns and villages." (nurse, mother of a nine-year-old child)

The interviews indicated that the reforms of health services in Bulgaria were accompanied by an insufficient information campaign. According to our respondents, it was not clear how people should access services, what services are provided for free, what co-payments are required from patients, where they need to be registered, and what documents they need to do so. People with low levels of education and no access to telephone or internet reportedly found it particularly difficult to find their way through the new system. Further challenges identified by our respondents were frequently changing lists of services and drugs covered by the NHIF:

"The bad thing is that almost every year the list of drugs and investigations and diseases that are covered by the Health Fund, which treatment is paid by the Health Fund, they change. Simply, today it is one thing, tomorrow it is something else, but the first one is no longer on the list and so on." (mother of a six-year-old child)

## Discussion

Although, following a qualitative research approach, we do not claim our study to be representative of users, providers and policy-makers concerned with child health services in Bulgaria, we believe that it points to a number of regulatory challenges associated with health reform in Central and Eastern Europe that warrant a closer and more systematic investigation that also captures changes over time (Table 2).

**Table 2:** Summary of key findings

Key regulatory failures	• Problems with health insurance • Informal payments • Insufficient regulation of the private sector
**Perceived causes of regulatory failures**	• Magnitude and pace of change • Lack of participation and sense of ownership of the reforms • "Commercialization" of medicine • Inadequate financing of the health system • Weak enforcement of regulations • Insufficient information about the health reforms

Over the last two decades, Bulgaria has shared with many other countries in Central and Eastern Europe the challenge of moving from a Soviet-style health system, in which the state was the main funder and provider of health services, to a more pluralist system with a variety of funding sources, including a health insurance system, and a stronger role of the private sector. This has brought with it an array of regulatory challenges, including how to establish a sustainable funding system, how to regulate private health care providers, and how to address burgeoning out-of-pocket payments and pharmaceutical expenditures [19,20].

The establishment of health insurance systems in Central and Eastern Europe has been particularly challenging [19,21,22]. In Bulgaria, one of the aims of introducing the health insurance system was to increase the level of funding available for health care. However, the NHIF failed to raise the expected revenues for a number of reasons and this has contributed to the failure of regulations to ensure equity in health care. The most important reason was the economic situation of the country, with low productivity and GDP. Second, there was a large number of people who were unable or unwilling to pay insurance contributions. According to official data, towards the end of 2007, the number of health-insured individuals in Bulgaria was 6,647,084, which meant that nearly 1 million people (or 13% of the population) were without health insurance coverage, with little change compared to previous years [23]. Socially disadvantaged groups, such as the Roma and the long-term unemployed, have been overrepresented among those without health insurance coverage [24,25]. Third, the percentage of wages allocated to health insurance (initially set at 6%) was low compared to insurance systems in other European countries [26]. In addition, a large number of people of working age are engaged in the informal "shadow" economy and avoid paying taxes and insurance contributions. A common practice among private employers is to declare officially a minimum salary for their employees so that insurance contributions are calculated on the basis of the minimum salary rather than the real wages which workers receive in cash. Nearly two thirds of insured persons in 2006 were insured at a level close to the minimum taxable salary [26]. Our study indicates that there might be a lack of motivation to contribute to the NHIF in view of the large scale of formal and informal payments at the point of use. These factors result in an unwillingness to participate in the solidarity system of health insurance and many people prefer to pay directly for services to public or private providers when they need health care. This practice undermines the pooling of risks and leads to problems of access to services, if uninsured people face unexpectedly high costs in case of serious illness [27].

Informal payments are a particular challenge to regulation [28]. While informal payments were reported in Bulgaria before 1998, when the Health Insurance Act was passed [5,16], the subsequent reforms in health financing, the introduction of user fees and new provider payment mechanisms do not seem to have eliminated such practices [16,29,30], and there are even indications that the share of out-of-pocket payments has increased [2]. According to a survey in January 2007, doctors were the occupational group from which respondents had most often been exposed to corruption pressure over the previous year [31]. Furthermore, charging user fees to exempted patients is one of the most commonly reported breaches in contracts of health care providers with the NHIF [32].

Increasingly, informal payments are perceived in the academic literature as a form of corruption [33]. Informal payments and other forms of corruption may deprive people of access to health care, and may lead to overpayments, inappropriate treatments and poor health outcomes. The extent of corruption also relates to the wider societal context. It is less likely in societies where there is general adherence to the rule of law, transparency and trust, where the public sector is accountable, and where the media and civil society are strong [34]. These are precisely the areas that are underdeveloped in many countries in Central and Eastern Europe [35,36] and there is now an extensive literature documenting the widespread nature of informal under-the-counter payments for health care in this part of Europe [34]. The low salaries of health workers in Central and Eastern Europe is a factor often mentioned in the literature on informal payments [31,37], a theme that resonated in the complaints of GPs in our study about having to treat children for a very low capitation fee. In view of the manipulations of user fees for outpatient services and their impact on access to services for vulnerable groups, there were plans to abandon them in 2009 [23], but these provoked protests among general practitioners [38].

However, regulatory failures go beyond the issue of informal payments. There are a number of other structural causes of providers' non-compliance with regulations. Frequent changes in payment mechanisms and the services which are partially or fully reimbursed by the NHIF contributed to an uncertain climate as to which co-payments were legal and which were not. This uncertainty was exacerbated by an insufficient information campaign surrounding the health reforms and increased the information asymmetry between patients and service providers. There is little evidence that the involvement of the population in health reforms has improved in recent years.

## Conclusions

Our findings suggest that major challenges facing the provision of health services in Bulgaria, such as the under-funding and limited coverage of the health insurance system or the existence of informal payments, can be partly understood as failures to enact and implement appropriate regulations. While doubts have been raised over the introduction of health insurance systems in countries such as Bulgaria [20], one reason why they have failed to reach their objectives is lacking adherence to regulations, underlining the need for better implementation. At the same time, an important finding of our study was that non-compliance with regulations is not only done for personal gain, but in some cases also for the perceived benefit of patients. Where regulations do make sense and are perceived as such by key actors of the health system, several policy measures could be considered to improve adherence. These include strengthening professional self-regulation, enforcing rules of good medical practice, revision of medical curricula with an emphasis on the ethics of the doctor-patient relationship, a reform of provider payment mechanisms, and ensuring quality improvements in service delivery. Overall, more attention needs to be paid by policy-makers to the regulatory frameworks, instruments and implementation mechanisms needed to accompany health reforms to ensure health services remain accessible to the population.

## List of abbreviations

EGN: Unique citizen's number; EU: European Union; GDP: Gross Domestic Product; GP: General Practitioner; NGO: Non-governmental Organization; NHIF: National Health Insurance Fund.

## Competing interests

The authors declare that they have no competing interests.

## Authors' contributions

BoR conceived of the study, carried out the fieldwork and drafted the manuscript. CB and NS participated in the planning of the study and the interpretation of results. BeR participated in the coordination of the study and the interpretation of the material and helped to draft the manuscript. All authors read and approved the final manuscript.

## Pre-publication history

The pre-publication history for this paper can be accessed here:

http://www.biomedcentral.com/1472-6963/11/219/prepub
